# Time-varying characteristics of remdesivir-treated patients hospitalised due to COVID-19: an electronic health record study

**DOI:** 10.7189/jogh.16.04038

**Published:** 2026-01-30

**Authors:** Jakob Kronkvist Hoe, Kim Blond, Espen Jimenez-Solem, Mark Berry, Mel Chiang, Mikkel Zöllner Ankarfeldt, Janne Petersen

**Affiliations:** 1 Bispebjerg and Frederiksberg Hospital, Copenhagen Phase IV unit (Phase4CPH) at department of Clinical Pharmacology and Centre of Clinical Research and Prevention, Copenhagen, Denmark; 2 Danish Medicines Council, Secretariat for Chronic Diseases, Copenhagen, Denmark; 3 Bispebjerg and Frederiksberg Hospital, Department of Clinical Pharmacology, Copenhagen, Denmark; 4 University of Copenhagen, Faculty of Health and Medical Sciences, Copenhagen, Denmark; 5 Gilead Sciences, Inc., Foster City, California, USA; 6 University of Copenhagen, Department of Public Health section of Biostatistics, Copenhagen, Denmark

## Abstract

**Background:**

Remdesivir is a pivotal antiviral treatment introduced during the COVID-19 pandemic, and its use has changed over time. This provided an opportunity to study how drug usage evolves over the course of a pandemic. Our study aimed to examine key physiological parameters of patients hospitalised due to COVID-19, how the characteristics of patients evolved, and explore if potential differences were similar across time for patients treated with remdesivir and those not treated.

**Methods:**

This study sourced electronic health care records from the Capital Region of Denmark. Patients aged ≥12 years hospitalised for the first time due to COVID-19 between 4 June 2020 and 1 December 2021 were included. Three time periods based on World Health Organization (WHO) treatment recommendations were used to describe temporal changes in the propensity score for remdesivir treatment as well as in individual patient characteristics.

**Results:**

In total, 6960 patients were included. The key differences between remdesivir-treated (n = 2557) and non-treated (n = 4403) were an elevated c-reactive protein (CRP) (median of 79 *vs*. 35 mg/L) and increased use of glucocorticoids (41.5% *vs*. 10%) and antithrombotics (48.5% *vs*. 18.5%). When describing the temporal changes in the propensity score, there was an overall significant interaction between the time period and exposure group. From the first to the middle period for the non-treated there was a significant increase in the mean propensity score of 0.04 (95% confidence interval (CI) = 0.02–0.06). The patient characteristics that had the largest temporal variations for remdesivir-treated patients were age, alanine transaminase, mechanical ventilation, interleukin-6 inhibitors, glucocorticoids, and antithrombotics.

**Conclusions:**

In conclusion, we found that remdesivir-treated and non-treated patients exhibited distinct sociodemographic and physiological characteristics. Also, the use of other COVID-19 treatments evolved differently between remdesivir-treated and not treated over time.

In recent years, COVID-19 has been one of the world’s largest global health crises [1]. Even though the World Health Organization (WHO) no longer declares it a pandemic, COVID-19 still poses a great risk to people with severe infections and health care sectors already under pressure [2,3]. Remdesivir, an RNA polymerase inhibitor approved for the treatment of COVID-19, was the first antiviral drug approved for the treatment of COVID-19 by Food and Drug Administration (FDA) in 2020 [4–6]. Over time the WHO recommendations for remdesivir use have varied. The use of remdesivir may also have been affected by virus mutations, a broader availability of other treatment options, a better understanding of remdesivir’s effect, and the emergence of vaccinations [7]. Furthermore, remdesivir holds the unique position of being one of the first approved treatments for and illness causing a global pandemic during an era of extensive data availability. Such a context provides a valuable opportunity to study how patient characteristics changed during the course of a pandemic for patients treated with remdesivir and those not treated.

Studies on remdesivir often involve different populations with varying comorbidities, and factors such as treatment timing, viral strain, and patient clinical status are believed to influence treatment outcomes of the individual study significantly [6,7]. These factors can mislead or introduce bias when interpreting or extrapolating results over time. Most of the major clinical randomised trials for remdesivir were conducted during the first year of the pandemic, leading to issues with generalisability when comparing these findings to patients treated later [8–12]. Subsequent observational studies on the effectiveness of remdesivir have compared the characteristics of remdesivir-treated *vs*. non-treated [13–27]. Some of these studies only reported the characteristics of a propensity score-matched sample [16,27]. Among those examining non-matched populations, some studies [13,15,17,21] found that remdesivir-treated patients had more severe COVID-19 than non-treated patients, while others observed the opposite trend [19,25,26]. To our knowledge, no studies have examined temporal changes in patient characteristics, including detailed clinical data, of patients with and without remdesivir treatment for COVID-19.

Given the dynamic nature of the COVID-19 pandemic, it is important to assess whether key clinical parameters of patients admitted with COVID-19 have remained consistent over time. It is natural to assume that the characteristics of admitted patients changed during the pandemic, and even more, that characteristics of patients treated with remdesivir changed over time. Describing these changes is essential to ensure that insights from the early use of remdesivir can be used to inform clinical decision-making in later use and perhaps even when a new pandemic arises. If the patients treated at the onset of the pandemic differ from those treated later, clinical decisions based on earlier data might be biased, necessitating more recent data to support decision-making. Furthermore, it can help guide further research by exploring which physical parameters have significantly changed since the early pandemic and thus needs to be accounted for to avoid bias when comparing remdesivir treatment with no treatment. The aim of this study is to describe key physiological parameters of patients hospitalised with COVID-19 stratified by remdesivir treatment. Furthermore, the study aims to describe how the characteristics of the remdesivir-treated population evolved over time and to describe if potential differences are similar across time for patients treated with remdesivir and those not treated.

## METHODS

### Study design, population, and study setting

Being a descriptive cohort study, electronic health care records are used to describe and compare the changes in the populations of COVID-19 patients treated with and without remdesivir over time. We included all individuals aged ≥12 years who were hospitalised and received their first hospital-based COVID-19 diagnosis (identified by ICD-10 codes) (Table S1 in the **Online Supplementary Document**) in the Capital Region of Denmark between 4 June 2020 and 1 December 2021. Exclusions were made for hospitalisations lasting less than four hours and where COVID-19 was not the primary diagnosis. Denmark is a country where access to health care is freely available to everyone and is paid through taxes. The Danish treatment guidelines for remdesivir closely followed the WHO recommendations regarding the use of remdesivir in the study period with a slight, one to five days, implementation delay [6,7]. There is a high degree of access to advanced medical treatments and high medical expertise [28], like many western countries. Data for the study was pseudonymised before it was made accessible to data processors.

### Data sources

The electronic health records from the Capital Region of Denmark were used to gather information on laboratory results, sociodemographics, drug prescriptions and administrations, physiological parameters, procedures, and diagnoses. Data was linked through a pseudo-anonymised, unique patient identifier number.

### Variables of interest

Exposure to remdesivir treatment was defined as the first administration of remdesivir following COVID-19 hospitalisation. It was identified via either the active ingredient (remdesivir) or the brand name (Veklury). Sociodemographic variables included age, sex, pregnancy, and smoking and were determined at the start of the COVID-19 hospitalisation.

Physiological parameters consisted of respiratory frequency, blood oxygen saturation, mean arterial blood pressure, body mass index (BMI), c-reactive protein (CRP), blood glucose, alanine aminotransferase (ALT), and estimated glomerular filtration rate. The earliest recorded value during COVID-19 hospitalisation was used. Other COVID-19 treatments, recorded within the first two days after hospitalisation, included the following: low-flow oxygen, high-flow oxygen/noninvasive ventilation (NIV), mechanical ventilation/extracorporeal membrane oxygenation (ECMO), interleukin-6 inhibitors, glucocorticoids, antithrombotics, convalescent plasma, and vasopressors, all identified through ATC and procedure codes (Table S2 in the **Online Supplementary Document**). Comorbidities were identified within two years prior to or on the date of COVID-19 hospitalisation. The following comorbidities were included: ischemic heart disease, kidney disease, cancer, immune deficiency, hypertension, diabetes, chronic lung disease, dementia, heart failure, atrial fibrillation, strokes, and chronic liver disease (Table S1 in the **Online Supplementary Document** for ICD-10 or ATC-codes).

In the following, the term ‘patient characteristics’ is used as an umbrella term encompassing sociodemographic variables, physiological parameters, other COVID-19 treatment, and comorbidities.

**Figure 1:**
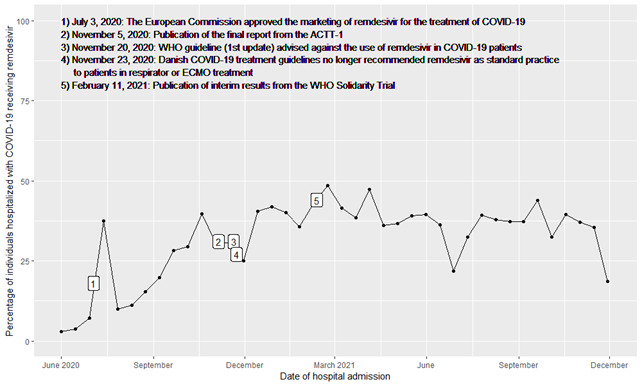
Percentage of individuals hospitalised with COVID-19 treated with remdesivir by calendar time with important events superimposed.

Time was separated into three time periods based on WHO’s recommendations for the use of remdesivir: from 4 June 2020 to 19 November 2020 (first time period); from 20 November 2020 to 10 February 2021 (middle time period); and from 11 February 2021 to 28 November 2021 (latest time period). On 3 June 2020, the European Commission approved the marketing of remdesivir for the treatment of COVID-19. Next, the WHO advised against remdesivir on 20 November 2020, due to interim results from the Solidarity and DisCoVeRy trials [7]. Finally, with the publication of newer trials such as PINETREE and the final results from Solidarity, remdesivir was again conditionally recommended [7,12,29] (**Figure 1**). Since the Danish treatment guidelines trailed one to five days after WHO recommendations, the time periods reflected the potential of treatment change. Patients were grouped based on these time periods according to the date of COVID-19 hospitalisation.

### Statistical analysis

To compare patient characteristics of remdesivir-treated and non-treated, Mann-Whitney U tests were used to test for differences in continuous variables. χ^2^ tests were used for categorical variables. The physiological parameters were affected by missing values. To address this issue, the missing values were imputed once for each patient using PROC MI in SAS, based on a missing at random approach. Each parameter was imputed individually and based on other available patient characteristics as well as time period and remdesivir exposure. The results of the complete case analyses will be presented in the supplementary material. A propensity score is often used to account for differences in patient characteristics between treatment groups in an outcome study. However, in the present study, we used a propensity score to simply describe patient characteristics in a single score. The propensity score was estimated using logistic regression with remdesivir treatment as the dependent variable and all patient characteristics as the predictors, except blood glucose due to high missingness. Continuous predictor variables (CRP, ALT, oxygen saturation, BMI, mean arterial blood pressure, respiratory frequency, and age) were modelled with restricted cubic splines with five knots positioned at the 5th, 27.5th, 50th, 72.5th and 95th percentiles of the variable, while eGFR was categorised into <30, 30–60, and ≥60 mL/min/1.73 m^2^. This results in a value ranging between 0 and 1, describing the propensity of being treated with remdesivir, based on the patient characteristics. Patients with a similar propensity score are comparable regarding patient characteristics, regardless of their actual treatment. The distribution of propensity scores across the time periods was visually presented in histograms. To test if the propensity score changed differently over time between the remdesivir treated and non-treated the interaction between time period and exposure group with the propensity score as the outcome in a linear regression was investigated. Furthermore, the mean difference in the propensity score over time was investigated, stratified on exposure group. To describe how patient characteristics differed, the mean or percentages and corresponding 95% confidence interval (CI) for the individual patient characteristic covariates were provided, stratified on time period and exposure group. Finally, to describe if individual patient characteristics impacted the probability of receiving remdesivir treatment differently over time, the interaction between time period and the patient characteristic was tested using logistic regression models with remdesivir use as the outcome. Statistical analyses were carried out using SAS enterprise guide 8.3 (Sas institute, Cary, North Carolina, USA).

## RESULTS

**Figure 2:**
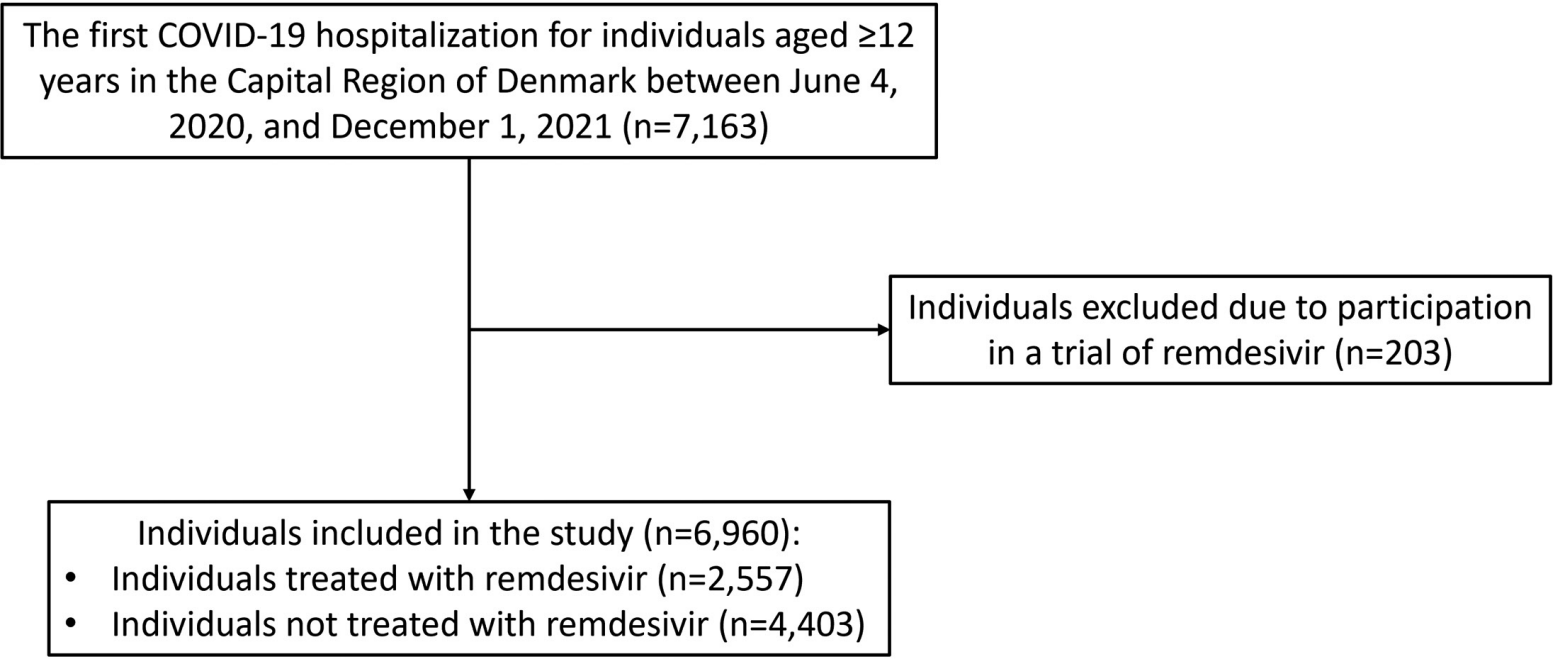
Flowchart of patients hospitalised due to COVID-19 in the Capital Region of Denmark.

Among 6960 individuals hospitalised due to COVID-19 between 4 June 2020 and 1 December 2021, 2557 individuals were treated with remdesivir (**Figure 2**). The majority started treatment shortly after hospitalisation: 76% within one day, 88% within three days, and 99% within 11 days.

The following sociodemographic characteristics differed significantly (*P* < 0.05) between remdesivir-treated and non-treated patients: remdesivir-treated patients were older (median of 65 *vs*. 60 years), less likely to be women (40% *vs*. 52%), and fewer treated were pregnant (0.5% *vs*. 1.5%) (**Table 1**). The following physiological parameters were significantly higher for the remdesivir-treated than for non-treated: median of non-mechanically ventilated respiration frequency (21 *vs*. 20 breaths per minute), BMI (28 *vs*. 26 kg/m^2^), CRP (79 *vs*. 35 mg/L), and ALT (31 *vs*. 28 U/L). The following physiological parameters were significantly lower for the remdesivir-treated than for non-treated: median oxygen saturation (94% *vs*. 97%) and eGFR (81 *vs*. 85 mL/min/1.73 m^2^). The use of other COVID-19 treatments was significantly higher in the remdesivir-treated group compared to non-treated in the following: low-flow oxygen (1.5% *vs*. 0.5%), high-flow oxygen (5% *vs*. 1%), mechanical ventilation/ECMO (9% *vs*. 3%), interleukin-6 inhibitors (3.5% *vs*. 0.5%), glucocorticoids (41.5% *vs*. 10%), and antithrombotic medication (48.5% *vs*. 18.5%). When comparing comorbidities between remdesivir-treated and non-treated, significantly fewer remdesivir-treated patients had kidney disease (2.5% *vs*. 7%), which was expected because low eGFR was contraindicated for treatment with remdesivir. Fewer remdesivir-treated patients had immune deficiency (1.5% *vs*. 2.5%), and more remdesivir-treated had cancer (9.5% *vs*. 7%) compared to non-treated.

**Table 1:** Characteristics of patients hospitalised due to COVID-19, stratified by remdesivir treatment, with missing physiological parameters imputed

Characteristics	No remdesivir (n = 4403)	Remdesivir (n = 2557)	
	MD (IQR) or n (%)*	MD (IQR) or n (%)*	***P*-value**†
**Sociodemographic**			
Age at the time of hospitalisation	60 (44, 77)	65 (51, 78)	<0.001
Women, n (%)	2288 (52%)	1023 (40%)	<0.001
Pregnant, n (%)	76 (1.5%)	10 (0.5%)	<0.001
Smoking (yes), n (%)	295 (6.5%)	192 (7.5%)	0.202
**Physiological parameters**			
Respiration frequency (breaths per minute)	20 (17, 23)	21 (18, 25)	<0.001
Oxygen saturation (%)	97 (94, 98)	94 (92, 96)	<0.001
Mean arterial blood pressure (mm Hg)	94 (85, 104)	94 (85, 103)	0.165
Body mass index (kg/m^2^)	26 (23, 31)	28 (24, 33)	<0.001
C-reactive protein (mg/L)	35 (11, 82)	79 (40, 136)	<0.001
Alanine aminotransferase (U/L)	28 (18, 48)	31 (20, 50)	<0.001
Estimated glomerular filtration rate (mL/min/1.73 m^2^)	85 (60, 90)	81 (61, 90)	0.004
**Other COVID-19 treatment**			
Low-flow oxygen, n (%)	18 (0.5%)	33 (1.5%)	<0.001
High-flow oxygen/NIV, n (%)	53 (1%)	129 (5%)	<0.001
Mechanical ventilation or ECMO, n (%)	125 (3%)	225 (9%)	<0.001
Interleukin-6 inhibitor, n (%)	19 (0.5%)	85 (3.5%)	<0.001
Glucocorticoids, n (%)	437 (10%)	1065 (41.5%)	<0.001
Antithrombotic medication, n (%)	807 (18.5%)	1240 (48.5%)	<0.001
Convalescent plasma, n (%)	15 (0.5%)	11 (0.5%)	0.555
Vasopressors, n (%)	51 (1%)	31 (1%)	0.840
**Comorbidity**‡			
Ischemic heart disease, n (%)	419 (9.5%)	239 (9.5%)	0.816
Kidney disease, n (%)	305 (7%)	67 (2.5%)	<0.001
Cancer, n (%)	315 (7%)	240 (9.5%)	<0.001
Immune deficiency, n (%)	111 (2.5%)	40 (1.5%)	0.008
Hypertension, n (%)	1774 (40.5%)	1214 (47.5%)	<0.001
Diabetes, n (%)	785 (18%)	678 (26.5%)	<0.001
Chronic lung disease, n (%)	926 (21%)	594 (23%)	0.032
Dementia, n (%)	195 (4.5%)	102 (4%)	0.382
Heart failure, n (%)	182 (4%)	111 (4.5%)	0.678
Atrial fibrillation, n (%)	382 (8.5%)	214 (8.5%)	0.659
Stroke, n (%)	211 (5%)	118 (4.5%)	0.737
Chronic liver disease, n (%)	68 (1.5%)	37 (1.5%)	0.748

ECMO – extracorporeal membrane oxygenation, IQR – interquartile range, MD – median, NIV – noninvasive ventilation

*Presented as median (IQR) with the exception of women, pregnant, smoking, other COVID-19 treatment, and comorbidities shown as n (%).

†Mann-Whitney U test was used to test for differences in continuous variables, and χ^2^ tests were used for categorical variables.

‡From two years before hospitalisation until and including the day of hospitalisation.

### Changes in propensity score over time

**Figure 3:**
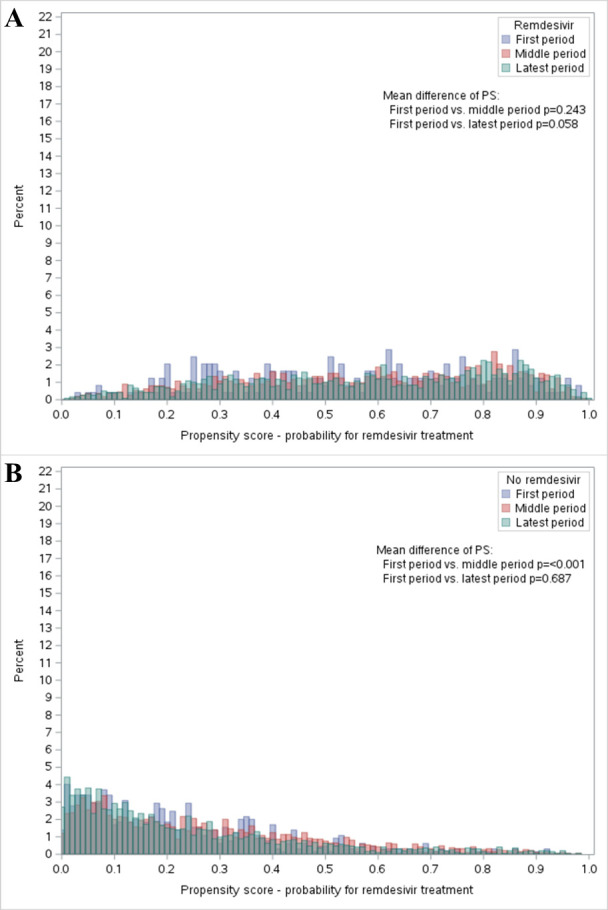
Histograms of the propensity scores of receiving remdesivir in the three time periods. **Panel A.** Distribution for remdesivir-treated. **Panel B.** Non-treated. The propensity scores were obtained from a logistic regression model that included sociodemographics, comorbidities, other COVID-19 treatment, and where missing, physiological parameters were imputed. *P*-values obtained using one-sided ANOVA. PS – propensity score.

The total change in all patient characteristics can generally be described by a change in the propensity score over time. Overall, the interaction between treatment period and exposure group suggested a difference in the propensity score (*P* < 0.001). The histograms show a large overlap in propensity score over time in both remdesivir-treated (**Figure 3**, panel A) and non-treated patients (**Figure 3**, panel B). The remdesivir-treated patients had a mean propensity score in the first period of 0.55 (95% CI = 0.52, 0.58). The mean change in propensity score from the first to the middle period was 0.02 (95% CI = −0.01, 0.05, *P* = 0.24). From the first to the latest period, there was an increase of 0.03 (95% CI = 0.00, 0.07, *P* = 0.058) in the mean propensity score. The non-treated patients in the first period had a mean propensity score estimate of 0.23 (95% CI = 0.21, 0.24). The change in the mean propensity score from the first to the middle period for the non-treated was an increase of 0.04 (95% CI = 0.02, 0.06, *P* < 0.001). From the first to the latest period, there was no change, 0.00 (95% CI = −0.02, 0.02, *P* = 0.687).

### Changes and interaction between patient characteristics, time, and treatment

The interaction between time period and patient characteristic with remdesivir use as the outcome was statistically significant for the following patient characteristics: age, sex, oxygen saturation, CRP, ALT, eGFR, mechanical ventilation/ECMO, glucocorticoids, antithrombotics, vasopressors, hypertension, and diabetes (**Table 2**). The changes in age were more extreme for the non-treated group, with an increase from the first, 60.0 (95% CI = 58.5, 61.5), to the middle period, 66.7 (95% CI = 65.8, 67.6), and a decrease from the middle to the latest period, 52.3 (95% CI = 51.4, 53.2). Oxygen saturation was stable over the three periods for the remdesivir-treated group, 93.4% (95% CI = 92.8, 94.0), 93.3% (95% CI = 93.0, 93.7), 93.3% (95% CI = 92.9, 93.6), whereas the non-treated group had a decrease from the first period, 96.6% (95% CI = 96.3, 96.9), to the middle period, 95.7% (95% CI = 95.5, 95.9). C-reactive protein was mostly consistent in the remdesivir group, with a mean of 94.3 mg/L (95% CI = 90.2, 98.5) to 97.7 mg/L (95% CI = 88.3, 107.0) but varied across time for the non-remdesivir group (**Table 2**). The use of mechanical ventilation/ECMO for the remdesivir treated decreased over the periods, from 14.0% (95% CI = 9.9, 19.0) to 7.0% (95% CI = 5.6, 8.6), whereas there was a more than 3-fold increase from the first period to the middle period for non-treated, 0.9% (95% CI = 0.3, 2.0) to 3.6% (95% CI = 2.8, 4.5). The treatment with glucocorticoids increased for remdesivir-treated over the periods from 34.2% (95% CI = 28.2, 40.5) to 45.2% (95% CI = 42.3, 48.1), whereas for non-treated it spiked in the middle period and returned to the level of the first period in the latest period. The use of antithrombotic medication for remdesivir non-treated had an increase from the first to the middle period, from 14.2% (95% CI = 11.6, 17.2) to 22.8% (95% CI = 20.9, 24.8) but remained rather stable over the three periods for remdesivir-treated (**Table 2**). For hypertension, a smaller reduction was seen from the middle to the latest period for remdesivir-treated patients compared to non-treated, 53.6% (95% CI = 50.6, 56.6) to 41.0% (95% CI = 38.2, 43.9) *vs*. 50.4% (95% CI = 48.1, 52.7) to 30.2% (95% CI = 28.1, 32.3). For diabetes a reduction was seen in the middle to the latest period among non-treated, from 20.1% (95% CI = 18.3, 22.0) to 15.0% (95% CI = 13.4, 16.6), not seen in the remdesivir-treated group (**Table 2**).

**Table 2:** Patient characteristics for each of the time periods for remdesivir-treated and non-treated with missing values for physiological parameters imputed

Characteristics	Remdesivir	First period*	Middle period*	Latest period*	*P*-value for interaction†
**Sociodemographic**					
Age	No	60.0 (58.5, 61.5)	66.7 (65.8, 67.6)	52.3 (51.4, 53.2)	0.001
	Yes	66.0 (64.1, 67.9)	68.8 (67.9, 69.7)	58.5 (57.5, 59.5)	
Women, % (95% CI)	No	55.3 (51.4, 59.2)	50.9 (48.6, 53.2)	51.9 (49.6, 54.1)	0.039
	Yes	34.6 (28.6, 40.9)	40.7 (37.8, 43.6)	40.5 (37.7, 43.3)	
Smoking, yes, % (95% CI)	No	7.9 (5.9, 10.2)	7.3 (6.1, 8.6)	5.7 (4.7, 6.9)	0.065
	Yes	4.5 (2.3, 8.0)	8.2 (6.7, 10.0)	7.4 (6.0, 9.1)	
**Physiological parameters**					
Respiration frequency (breaths per minute)	No	19.7 (19.4, 20.1)	20.5 (20.2, 20.7)	20.2 (20.0, 20.5)	0.486
	Yes	22.3 (21.3, 23.2)	22.7 (22.3, 23.0)	22.6 (22.3, 23.0)	
Oxygen saturation (%)	No	96.6 (96.3, 96.9)	95.7 (95.5, 95.9)	96.2 (96.0, 96.4)	<0.001
	Yes	93.4 (92.8, 94.0)	93.3 (93.0, 93.7)	93.3 (92.9, 93.6)	
Mean arterial blood pressure (mm Hg)	No	94.8 (93.8, 95.9)	95.5 (94.8, 96.2)	93.9 (93.2, 94.6)	0.218
	Yes	93.2 (91.4, 94.9)	94.8 (93.9, 95.7)	94.1 (93.3, 94.9)	
Body mass index (kg/m^2^)	No	27.3 (26.8, 27.8)	26.6 (26.3, 26.9)	27.2 (26.9, 27.5)	0.268
	Yes	28.7 (28.0, 29.5)	28.1 (27.7, 28.5)	29.7 (29.1, 30.4)	
C-reactive protein (mg/L)	No	44.7 (40.6, 48.7)	65.9 (62.7, 69.1)	53.1 (50.4, 55.9)	<0.001
	Yes	97.7 (88.3, 107)	99.9 (95.6, 104)	94.3 (90.2, 98.5)	
Alanine aminotransferase (U/L)	No	51.3 (45.8, 56.8)	50.5 (45.7, 55.3)	56.0 (52.5, 59.4)	<0.001
	Yes	40.5 (34.3, 46.8)	53.7 (25.3, 82.1)	46.5 (43.9, 49.1)	
Estimated glomerular filtration rate (mL/min/1.73m^2^)	No	73.3 (71.4, 75.1)	66.4 (65.2, 67.6)	78.1 (77.1, 79.0)	<0.001
	Yes	71.7 (69.2, 74.3)	70.8 (69.7, 72.0)	76.8 (75.8, 77.8)	
**Other COVID-19 treatment**					
Low-flow oxygen, % (95% CI)	No	0.2 (0.0, 0.9)	0.3 (0.1, 0.7)	0.6 (0.3, 1.0)	0.530
	Yes	Less than 3	1.5 (0.9, 2.4)	1.3 (0.8, 2.2)	
High-flow oxygen/NIV, % (95% CI)	No	0.8 (0.3, 1.8)	1.4 (0.9, 2.0)	1.2 (0.8, 1.8)	0.567
	Yes	5.3 (2.9, 9.0)	5.1 (3.9, 6.5)	4.9 (3.8, 6.3)	
Mechanical ventilation or ECMO, % (95% CI)	No	0.9 (0.3, 2.0)	3.6 (2.8, 4.5)	2.8 (2.1, 3.6)	<0.001
	Yes	14.0 (9.9, 19.0)	9.6 (7.9, 11.4)	7.0 (5.6, 8.6)	
Interleukin-6 inhibitor, % (95% CI)	No	Less than 3	Less than 3	1.0 (0.6, 1.5)	0.962
	Yes	Less than 3	0.2 (0.0, 0.6)	6.9 (5.6, 8.5)	
Glucocorticoids, % (95% CI)	No	6.6 (4.9, 8.8)	11.1 (9.7, 12.6)	9.9 (8.6, 11.3)	0.024
	Yes	34.2 (28.2, 40.5)	39.5 (36.6, 42.4)	45.2 (42.3, 48.1)	
Antithrombotic medication, % (95% CI)	No	14.2 (11.6, 17.2)	22.8 (20.9, 24.8)	15.4 (13.9, 17.1)	<0.001
	Yes	44.9 (38.5, 51.3)	45.7 (42.7, 48.6)	51.9 (49.0, 54.7)	
Vasopressors, % (95% CI)	No	0.5 (0.1, 1.3)	1.5 (1.0, 2.2)	1.0 (0.6, 1.6)	0.050
	Yes	2.1 (0.7, 4.7)	1.0 (0.5, 1.8)	1.3 (0.7, 2.1)	
**Comorbidity**‡					
Ischemic heart disease, % (95% CI)	No	10.2 (8.0, 12.8)	11.1 (9.7, 12.7)	7.7 (6.6, 9.0)	0.259
	Yes	7.0 (4.1, 11.0)	10.8 (9.1, 12.8)	8.5 (6.9, 10.2)	
Kidney disease, % (95% CI)	No	6.3 (4.6, 8.5)	8.9 (7.6, 10.3)	5.3 (4.3, 6.4)	0.784
	Yes	2.9 (1.2, 5.8)	3.1 (2.2, 4.3)	2.1 (1.4, 3.1)	
Cancer, % (95% CI)	No	6.3 (4.6, 8.5)	9.2 (8.0, 10.7)	5.4 (4.5, 6.5)	0.123
	Yes	6.2 (3.5, 10.0)	10.8 (9.1, 12.8)	8.7 (7.2, 10.4)	
Immune deficiency, % (95% CI)	No	1.7 (0.9, 3.0)	2.3 (1.7, 3.1)	3.0 (2.3, 3.9)	0.643
	Yes	Less than 3	1.8 (1.1, 2.7)	1.7 (1.0, 2.6)	
Hypertension, % (95% CI)	No	41.6 (37.7, 45.5)	50.4 (48.1, 52.7)	30.2 (28.1, 32.3)	0.005
	Yes	51.0 (44.6, 57.5)	53.6 (50.6, 56.6)	41.0 (38.2, 43.9)	
Diabetes, % (95% CI)	No	19.9 (16.9, 23.2)	20.1 (18.3, 22.0)	15.0 (13.4, 16.6)	0.049
	Yes	24.3 (19.0, 30.2)	27.9 (25.3, 30.6)	25.7 (23.2, 28.3)	
Chronic lung disease, % (95% CI)	No	21.9 (18.8, 25.3)	22.7 (20.8, 24.7)	19.1 (17.4, 20.9)	0.250
	Yes	19.3 (14.6, 24.9)	25.2 (22.7, 27.9)	22.2 (19.8, 24.6)	
Dementia, % (95% CI)	No	3.7 (2.4, 5.5)	7.8 (6.6, 9.2)	1.4 (0.9, 2.0)	0.245
	Yes	2.9 (1.2, 5.8)	6.4 (5.1, 8.0)	1.9 (1.2, 2.9)	
Heart failure, % (95% CI)	No	5.4 (3.8, 7.4)	5.1 (4.2, 6.2)	2.8 (2.1, 3.6)	0.578
	Yes	4.5 (2.3, 8.0)	5.3 (4.0, 6.7)	3.4 (2.5, 4.6)	
Atrial fibrillation, % (95% CI)	No	8.8 (6.7, 11.3)	12.1 (10.7, 13.7)	5.3 (4.4, 6.4)	0.771
	Yes	7.4 (4.4, 11.5)	12.2 (10.4, 14.3)	4.9 (3.8, 6.3)	
Stroke, % (95% CI)	No	3.1 (1.9, 4.7)	7.2 (6.1, 8.5)	3.0 (2.3, 3.9)	0.248
	Yes	3.3 (1.4, 6.4)	5.9 (4.6, 7.4)	3.7 (2.7, 4.9)	
Chronic liver disease, % (95% CI)	No	1.1 (0.4, 2.2)	1.8 (1.2, 2.5)	1.5 (1.0, 2.1)	0.433
	Yes	2.1 (0.7, 4.7)	1.5 (0.9, 2.4)	1.3 (0.7, 2.1)	

CI – Confidence interval, ECMO – extracorporeal membrane oxygenation, NIV – noninvasive ventilation

*The columns ‘First period’, ‘Middle period’, and ‘Latest period’ are presented as ‘mean (95% CI)’ unless otherwise stated.

†The *P*-value for the interaction is based on a logistic regression model with remdesivir treatment as the outcome and the interaction between the variable and the time period.

‡From two years before hospitalisation until and including the day of hospitalisation.

## DISCUSSION

In this study of 6960 COVID-19 patients, remdesivir was typically administered to those with lower oxygen saturation and higher CRP levels compared to non-treated patients, indicating more severe illness. The prevalence of chronic kidney disease and immune deficiency was lower among remdesivir-treated than non-treated, whereas the opposite was the case for cancer. Remdesivir-treated patients were more often men and older. To describe changes over time, we compared the mean propensity score and individual patient characteristics across time for remdesivir-treated and non-treated patients separately. The statistically significant interaction term between time period and exposure group suggests that characteristics of the exposure groups evolved differently over time. No change in the mean propensity score was seen for remdesivir-treated across the three time periods. Among remdesivir treated the individual characteristics with the largest differences were a reduction in age and in the use of mechanical ventilation/ECMO, and an increased use of other COVID-19 medicinal treatments. For the non-treated group, there was a significant increase in the mean propensity score from the first to the middle period but no significant change from the first to the latest period. The interaction analyses of each of the individual patient characteristic showed that age, sex, oxygen saturation, CRP, ALT, eGFR, mechanical ventilation/ECMO, glucocorticoids, antithrombotic medication, vasopressors, hypertension, and diabetes interaction with time period changed the probability of receiving remdesivir.

In the complete case analysis, the mean propensity score is higher for both exposure groups and the changes of the propensity score between time periods larger (Table S3–7 and Figure S1 in the **Online Supplementary Document**). The probability increase was larger in the no-treatment group compared to the treatment group and change in propensity score in the latest period more pronounced. This difference in results, whether propensity score analysis was based on complete case or imputation, aligned with the authors’ expectations. Missing data was expected due to medical staff not testing the most healthy and non-severe patients. With imputation the number of healthier patients thus increase, which will lower the propensity score, especially among subgroups with higher missingness such as non-treated and latest time period.

In studies of the effectiveness or safety of remdesivir, some [13,15,17,21] found that remdesivir-treated patients had more severe COVID-19 than non-treated, and some found the opposite pattern [19,25,26]. Other studies did not find a clear pattern [18,22] or did not examine COVID-19 severity adjacent to remdesivir administration [14,23,30]. The different findings may be explained by the specific study context, *e.g*. out-patient setting [23,30], intensive care setting [19], exclusion of immune-compromised patients [21], restricting to pregnant women [24], and historical comparators [26]. As such, differences in the characteristics of remdesivir-treated and non-treated appear to be very context-specific. Our study includes all patients hospitalised due to COVID-19 without including or excluding specific diseases, focusing on all patients possible to treat with remdesivir, thus offering a more complete picture. Furthermore, the Danish COVID-19 treatment guidelines included more specific treatment initiation criteria than the product information [31], which could explain some of the differences between studies. The higher prevalence of kidney disease observed in the non-treated group is likely explained by the remdesivir product information stating at the time that remdesivir should not be used in individuals with eGFR<30 ml/min/1.73 m^2^.

Other COVID-19 medication was so far only reported in a few other studies [21,22,25]. Other COVID-19 medication use in our study was higher in the remdesivir group, except for vasopressors, which were equally uncommon, 1%, across groups. Panda et al. [21] found higher, 39.5% vasopressor use in the remdesivir group compared to the control (28.6%). The large disparity between studies might be explained by Panda et al. focusing on major adverse cardiac events, leading to sampling selection with a high risk of vasopressor use. Pilgram et al. found a non-significant difference in the use of convalescent plasma, anticoagulants, and interleukin-6 inhibitors but increased steroid use in the non-remdesivir group [22]. We report the same findings regarding convalescent plasma, most likely driven by the low number of patients receiving the treatment. We found significantly more use of anticoagulants, interleukin-6 inhibitors, and steroids in the remdesivir group, which is opposed to the findings of Pilgram et al. The LEOSS database, which Pilgram’s study is based on, contains 136 different sites and roughly 11 000 patients. The sites manually and voluntarily report the cases through an electronic case report form to the database. This could lead to selection bias if only a select group of patients were voluntarily reported. This is not the case in our study since all data are gathered automatically. Tsuzuki et al. included only non-severe COVID-19 patients in their study but found that 80.8% of patients treated with remdesivir were also treated with steroids, compared to 15.8% of controls, and 39.7% *vs*. 26% were treated with interleukin-6 inhibitors. [25]. Our study found similar trends regarding other COVID-19 medication, although proportions of steroid use were different, with 41.5% in the remdesivir group *vs*. 10% in the non-treated group. Since the remdesivir-treated group had more severe COVID-19, we expected to see this increased steroid use due to Danish guidelines not recommending steroids for non-severe COVID-19 infections [32].

Though the current study is designed to be purely descriptive, the following paragraph will discuss some possible setting specific factors that could have impacted the divergence over time. The drop in the use of remdesivir for patients in mechanical ventilation/ECMO treatment is likely explained by changes to the Danish COVID-19 treatment guidelines. On 23 November 2020, remdesivir was excluded as the standard practice for patients in respirator or ECMO treatment [31]. This aligns with the observation in our study: a reduction from 14.0% to 9.6% between the first and middle period. Data on vaccination was not available in the present study. However, with the emergence of vaccinations, older adults – being at higher risk of severe COVID-19 – gained the most protection, leading to fewer older adults among hospitalised patients and thus a decline in age among patients admitted with COVID-19 could be expected. A drop in age for both remdesivir-treated and non-treated from the middle period to the latest period was observed, though more extreme for non-treated. Vaccinations became available in Denmark on 27 December 2020, and were prioritised for the elderly and high-risk patients. By the start of the latest period, 19 February 2021, 7.84% of the Danish population had received one or more vaccinations and by the end of the latest period 29 November 2021, 75.8% had received at least two vaccinations [33]. Implementation of vaccination could thus explain the reported decline in age and might also explain what is observed with hypertension and diabetes since both are often correlated with age.

An important finding of this study is the change over time in practices regarding other COVID-19 treatments alongside remdesivir. An increase in the use of glucocorticoids (34.2% to 45.2%) and antithrombotics (44.9% to 51.9%) was only observed in the remdesivir-treated group. The increase in the use of antithrombotics was not driven by an increase in heart disease-related comorbidity. While increased other COVID-19 medication use may not affect the intention to treat effect of remdesivir examined through clinical trials, it will most likely change the effectiveness of COVID-19 treatment as a whole and thus impact the effectiveness of remdesivir in a real-world setting. It is expected that combination treatment with remdesivir and glucocorticoids or antithrombotics improves the overall survival rates of patients admitted with COVID-19. Therefore, failing to adjust for the impact of combination therapy could lead to an overestimation of remdesivir’s effectiveness.

The significant interaction of time period and exposure group on propensity score suggests a different development over time between remdesivir-treated and non-treated. Based on the propensity score remdesivir-treated patients did not differ from each other across time periods, while this was the case for non-treated patients. Temporal divergence between corresponding exposure groups in clinical trials could be one the factors in diverging trial results over calendar time. Comparison of trial results at differing points in calendar time need to account for diverging developments in patient characteristics between exposure groups if present. Furthermore, since randomised clinical trials often do not include other COVID-19 medication that affect the outcome, the effectiveness of a drug in a real-world setting could diverge from the effect found in trials. Along with new COVID-19 variants of concern and the availability of vaccinations, this strongly suggests an unmet need for new effectiveness studies of remdesivir that account for other COVID-19 treatments, variants of concern, and vaccination.

### Limitations

Our study comes with limitations. The study results may be difficult to generalise internationally, despite the WHO's global guideline changes, as the COVID-19 situation was unique to each country, with the possibility of national guidelines superseding international ones. In the Danish health care setting the national guidelines closely followed WHO recommendations.

The physiological parameters represent a single time point. To be able to compare treated and not treated, a similar time point across remdesivir-treated and non-treated is needed. We therefore selected the first measurement after hospitalisation, regardless of remdesivir initiation, since requiring measurements to be taken before remdesivir initiation would naturally only be feasible in the remdesivir-treated group. Ideally, the measurements are taken before remdesivir, so that they do not reflect benefit from treatment. Among remdesivir-treated 76% initiated remdesivir within one day and 88% within three days. Often physiological parameters are assed when patients are just admitted to the hospital, and these values contribute to treatment decisions, which make first recorded value a reasonable choice.

Another limitation of this study is the lack of information on vaccinations. Future studies may look into how vaccination across treatment groups changed over time. The first vaccination in Denmark occurred on 27 December 2020, within the time frame of this study, but data on vaccination could not be included due to data availability issues.

The study is purely descriptive and offers no explanations to why the groups diverged over time. Follow-up studies including major temporal factors such as changing viral strains, variants of concern, evolving guidelines, changes to health care settings, COVID-19 lockdown, and drug availability are needed to elucidate the reasons for the observed temporal divergence. Furthermore, it would be useful to study if the temporal divergences affect treatment effects on clinical outcomes, such as death and ICU admissions.

Although other COVID-19 treatments, including medication, are described in the present study they are not temporally sequenced. This means treatments could be either combination treatment with remdesivir or with each other, and we do not know how this affects the health status of the patients.

Many different patient characteristics have been investigated for interactions with time. No power calculations were performed and no adjustment for multiple testing was performed, therefore all results should be considered as explorative and purely descriptive.

## CONCLUSIONS

In conclusion, it was observed how remdesivir-treated and non-treated patients exhibited distinct sociodemographic and physiological characteristics. The use of COVID-19 treatments varied alongside remdesivir treatment, with other COVID-19 treatments evolving continuously. Notably, the differences between remdesivir-treated and non-treated patients were most pronounced during the first five months after the introduction of remdesivir in the COVID-19 treatment recommendations. The observed temporal divergence in characteristics between treated and non-treated suggests an unmet need for more recent data to be able to study the effectiveness of remdesivir. Likewise, temporal divergence is important to consider in future studies when investigating other treatments introduced in a rapidly changing pandemic.

## Additional material


Online Supplementary Document
