# Supplementary material for: Time-varying characteristics of remdesivir-treated patients hospitalised due to COVID-19: an electronic health record study
Source: J Glob Health. 2026 Jan 30;16:04038. doi: 10.7189/jogh.16.04038 (PMC12856963; doi:10.7189/jogh.16.04038)
Supplement: Online Supplementary Document [file jogh-16-04038-s001.pdf]

Supplement to: Hoe JK, Blond K, Jimenez-Solem E, Berry M, Chiang M, Ankarfeldt MZ, Petersen J. Time-varying characteristics of remdesivir-treated patients hospitalised due to COVID-19: an electronic health record study. J Glob Health. 2026;16:04038.

Table S1. Codes to identify comorbidities in patients hospitalized due to COVID-19

Disease	ICD-10 codes	ATC codes
COVID-19	B342, B342A, B972, B972A.	None
Ischemic heart disease	I20-I25	N02BA, C01DA, B01AC24
Hypertension	I10-I15	C08, C03A, C07, C09
Heart failure	I099A, I110, I130, I132, I50	None
Atrial fibrillation	I48	None
Stroke	I60-I64, I69	None
Diabetes	E10-E14	A10
Chronic lung disease	J41-47	R03AK, R03AL, R03BA, R03AC12, R03AC13, R03AC18, R03AC19, R03CC12, R03BB04-R03BB07
Dementia	F00-F03, F1073, F1173, F1273, F1373, F1473, F1573, F1673, F1873, F1973	N06D
Chronic liver disease	K700-K704, K709, K71-K74, K760, K766, B150, B160, B162, B18, B190, I85	None
Kidney disease	I12, I13, N00-N05, N07, N08, N11, N14, N18, N19, E102, E112, E142	None
Cancer	C00-97 except C44	None
Immune deficiency	D80-89, T86, Y830, Z94, B20, B-21-B24 with CD4 count<200 recorded during COVID-19 hospitalization	None

Table S2. Codes to identify treatments for patients hospitalized due to COVID-19

Treatment	ATC codes	Procedure codes
Remdesivir	J05AB16	None
Low-flow oxygen	None	BGXA5, BGXA50-BGXA57
High-flow oxygen	None	BGDA2
NIV	None	BGDA1
Ventilation or ECMO	None	BGXA2, KFXE, KFXE00
Interleukin-6 inhibitor (tocilizumab)	L04AC07	BOHJ18B2
Glucocorticoids	H02AB01-H02AB17	None
Antithrombotic medication (heparin group, platelet aggregation inhibitors excl. heparin, enzymes, direct thrombin inhibitors, direct factor Xa inhibitors, other antithrombotic agents)	BA01AA, B01AB01- B01AB12, B01AB51, B01AC01- B01AC30, B01AD01-B01AD12, B01AE01-B01AE07, B01AF01-B01AF04, B01AX01, B01AX04, B01AX05, B01AX07	None
Janus kinase inhibitors (baricitinib, ruxolitinib, tofacitinib)	L04AA37, L01EJ01, L04AA29	BWHA418, BOHJ28D
Convalescent plasma	None	BOQA0, BOQA00-BOQA05

Table S3. Characteristics of patients hospitalized due to COVID-19, stratified by remdesivir treatment with complete case dataset.

Characteristics	No remdesivir		Remdesivir		
	Number of observations	Median (IQR) or n (%)*	Number of observations	Median (IQR) or n (%)*	P-value†
**Sociodemographic**	**4,403**		**2,557**		
Age at the time of hospitalization		60 (44, 77)		65 (51,78)	<0.001
Women, n (%)		2,288 (52%)		1,023 (40%)	<0.001
Pregnant, n (%)		76 (1.5%)		10 (0.5%)	<0.001
Smoking (yes), n (%)		295 (6.5%)		192 (7.5%)	0.202
**Physiological parameters**					
Respiration frequency (breaths per minute)	3,160	20 (17,22)	2,496	21 (18,25)	<0.001
Oxygen saturation (%)	3,176	96 (95,98)	2,509	94 (92,96)	<0.001
Mean arterial blood pressure (mm Hg)	3,164	94 (85,103)	2,504	94 (85,103)	0.326
Body mass index (kg/m2)	3,255	26 (23,30)	2,247	28 (24,32)	<0.001
C-reactive protein (mg/L)	4,149	33 (10,79)	2,557	79 (40,136)	<0.001
Blood glucose (mmol/L)	1,572	7 (6,8)	1,822	7 (6,8)	<0.001
Alanine aminotransferase (U/L)	4,043	26 (18,42)	2,554	31 (20,50)	<0.001
Estimated glomerular filtration rate (mL/min/1.73 m2)	4,117	85 (58,90)	2,556	81 (61,90)	0.043
**Other COVID-1G treatment**	**4,403**		**2,557**		
Low-flow oxygen, n (%)		18 (0.5%)		33 (1.5%)	<0.001
High-flow oxygen/NIV, n (%)		53 (1%)		129 (5%)	<0.001
Mechanical ventilation or EMCO, n (%)		125 (3%)		225 (9%)	<0.001
Interleukin-6 inhibitor, n (%)		19 (0.5%)		85 (3.5%)	<0.001
Glucocorticoids, n (%)		437 (10%)		1,065 (41.5%)	<0.001
Antithrombotic medication, n (%)		807 (18.5%)		1,240 (48.5%)	<0.001
Convalescent plasma, n (%)		15 (0.5%)		11 (0.5%)	0.555
Vasopressors, n (%)		51 (1%)		31 (1%)	0.840
**Comorbidity‡**	**4,403**		**2,557**		
Ischemic heart disease, n (%)		419 (9.5%)		239 (9.5%)	0.816
Kidney disease, n (%)		305 (7%)		67 (2.5%)	<0.001
Cancer, n (%)		315 (7%)		240 (9.5%)	<0.001
Immune deficiency, n (%)		111 (2.5%)		40 (1.5%)	0.008
Hypertension, n (%)		1,774 (40.5%)		1,214 (47.5%)	<0.001
Diabetes, n (%)		785 (18%)		678 (26.5%)	<0.001
Chronic lung disease, n (%)		926 (21%)		594 (23%)	0.032
Dementia, n (%)		195 (4.5%)		102 (4%)	0.382
Heart failure, n (%)		182 (4%)		111 (4.5%)	0.678
Atrial fibrillation, n (%)		382 (8.5%)		214 (8.5%)	0.659
Stroke, n (%)		211 (5%)		118 (4.5%)	0.737
Chronic liver disease, n (%)		68 (1.5%)		37 (1.5%)	0.748

Notes:
*Presented as median (IQR) with the exception of women, pregnant, smoking, other COVID-19 treatment, and comorbidities shown as n (%).
†Mann-Whitney U test was used to test for differences in continuous variables, and chi-squared tests were used for categorical variables.
‡From 2 years before hospitalization until and including the day of hospitalization.
IQR – Interquartile range

Table S4. Amount and percentage of patients with at least one missing value of physical parameters used for propensity score model

	First period		Middle period		Latest period	
	N	%	N	%	N	%
No remdesivir	239	36.94%	614	33.39%	1,139	59.42%
Remdesivir	15	6.17%	119	10.63%	233	19.50%

Table S5. Overall test for the interaction of time period and exposure group on propensity score to receive remdesivir

P-value	Dataset
<0.001	Imputed
0.148	Complete case

Table S6. Mean propensity score to receiving remdesivir stratified by exposure group and dataset based on patient characteristics.

Time periods	Exposure group	Mean PS*	G5% CI	P-value†
**Complete case**				
First	Remdesivir	0.61	0.58-0.63	
First to middle	Remdesivir	0.00	-0.03-0.03	0.867
First to latest	Remdesivir	0.04	0.01-0.07	0.023
First	No remdesivir	0.31	0.29-0.33	
First to middle	No remdesivir	0.04	0.01-0.06	0.005
First to latest	No remdesivir	0.06	0.03-0.09	<0.001
**Imputed**				
First	Remdesivir	0.55	0.52-0.58	
First to middle	Remdesivir	0.02	-0.01-0.05	0.243
First to latest	Remdesivir	0.03	0-0.07	0.058
First	No remdesivir	0.23	0.21-0.24	
First to middle	No remdesivir	0.04	0.02-0.06	<0.001
First to latest	No remdesivir	0.00	-0.02-0.02	0.687

Notes:
*For time periods different from the first period, the mean propensity score is the change in

propensity score from the first period to the middle or latest.
†P-value is obtained using one-sided ANOVA test.
CI – confidence interval
PS – Propensity score

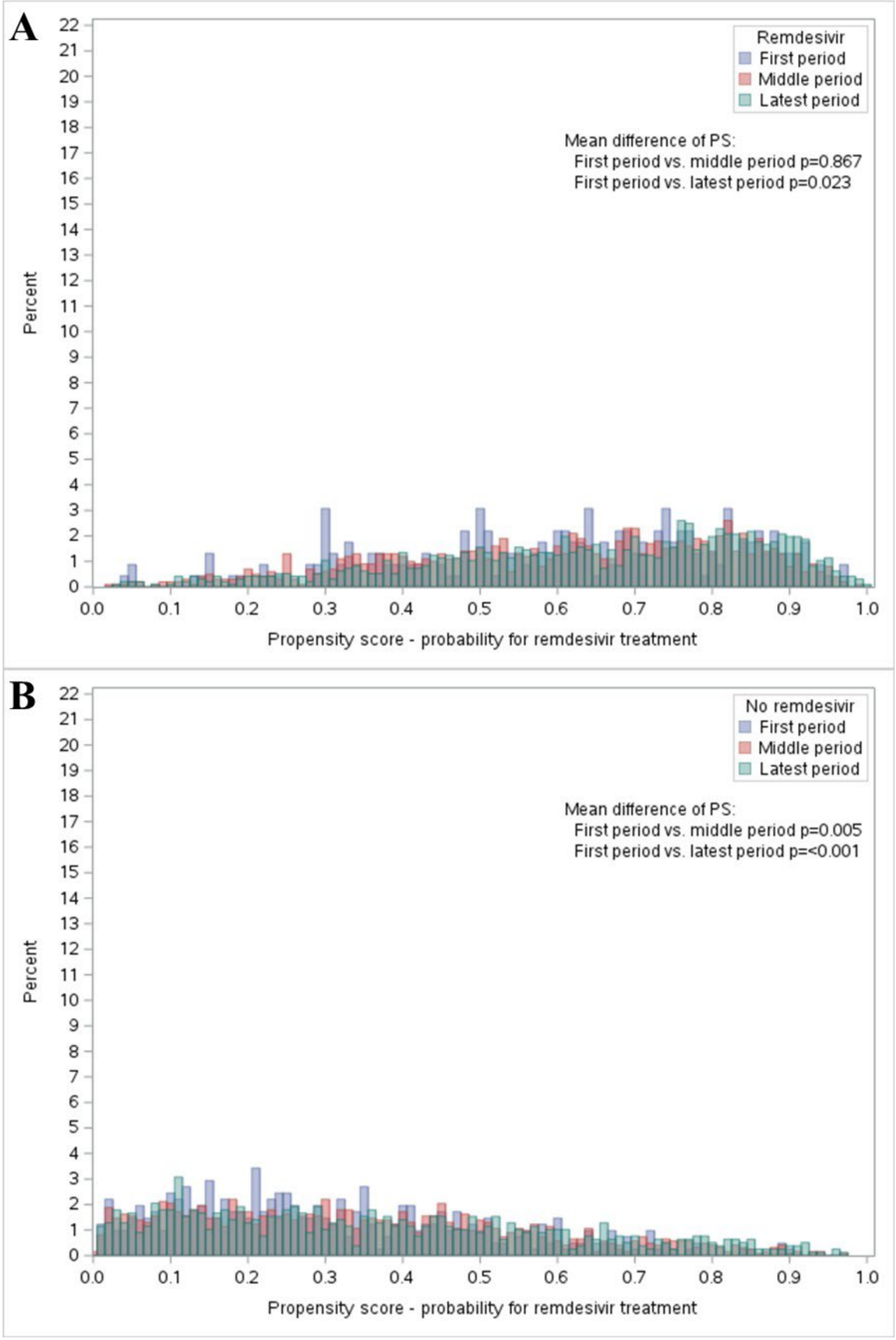

Figure S1. Histograms of the propensity scores of receiving remdesivir in the three time periods using complete case dataset. **Panel A** displays the distribution for remdesivir-treated and **Panel B** the non-treated. The propensity scores were obtained from a logistic regression model that included sociodemographics, comorbidities, other COVID-19 treatment, and

where missing, physiological parameters were imputed. P-values obtained using one-sided ANOVA. PS – propensity score.

Table S7. Patient characteristics for each of the time periods for remdesivir-treated and non-treated with complete case values.

Characteristics	Remdesivir	First period*	Middle period*	Latest period*	P-value for interaction †
**Sociodemographic**					
Age	No	60.0 (58.5-61.5)	66.7 (65.8-67.6)	52.3 (51.4-53.2)	0.001
	Yes	66.0 (64.1-67.9)	68.8 (67.9-69.7)	58.5 (57.5-59.5)	
Women, % (95% CI)	No	55.3 (51.4-59.2)	50.9 (48.6-53.2)	51.9 (49.6-54.1)	0.039
	Yes	34.6 (28.6-40.9)	40.7 (37.8-43.6)	40.5 (37.7-43.3)	
Smoking, yes, % (95% CI)	No	7.9 (5.9-10.2)	7.3 (6.1-8.6)	5.7 (4.7-6.9)	0.065
	Yes	4.5 (2.3-8.0)	8.2 (6.7-10.0)	7.4 (6.0-9.1)	
**Physiological parameters**					
Respiration frequency (breaths per minute)	No	19.8 (19.5-20.2)	20.6 (20.4-20.9)	20.5 (20.2-20.8)	0.363
	Yes	22.3 (21.3-23.2)	22.6 (22.3-23.0)	22.6 (22.2-23.0)	
Oxygen saturation (%)	No	96.5 (96.3-96.8)	95.5 (95.3-95.8)	95.8 (95.6-96.1)	<0.001
	Yes	93.4 (92.8-94.0)	93.3 (93.0-93.7)	93.3 (93.0-93.6)	
Mean arterial blood pressure (mm Hg)	No	94.8 (93.6-95.9)	95.4 (94.6-96.2)	93.8 (92.9-94.7)	0.262
	Yes	93.2 (91.4-94.9)	94.8 (93.9-95.7)	94.1 (93.3-94.9)	
Body mass index (kg/m2)	No	27.7 (27.2-28.2)	26.8 (26.4-27.1)	27.3 (27.0-27.6)	0.053
	Yes	28.7 (28.0-29.5)	28.0 (27.6-28.4)	29.8 (29.0-30.5)	
C-reactive protein (mg/L)	No	43.1 (38.9-47.4)	65.2 (61.9-68.5)	52.1 (49.2-55.0)	<0.001
	Yes	97.7 (88.3-107)	99.9 (95.6-104)	94.3 (90.2-98.5)	
Blood glucose (mmol/L)	No	7.2 (6.9-7.5)	7.3 (7.1-7.4)	7.1 (7.0-7.3)	0.777
	Yes	7.9 (7.5-8.3)	7.7 (7.6-7.9)	7.6 (7.4-7.7)	
Alanine aminotransferase (U/L)	No	36.5 (33.4-39.6)	40.1 (35.7-44.6)	40.7 (38.7-42.6)	0.003
	Yes	40.5 (34.3-46.8)	53.7 (25.3-82.1)	46.6 (44.0-49.1)	
Estimated glomerular filtration rate	No	72.9 (71.0-74.8)	65.7 (64.5-67.0)	77.4 (76.4-78.4)	<0.001
(mL/min/1.73m^2)	Yes	71.7 (69.2-74.3)	70.8 (69.7-72.0)	76.8 (75.8-77.8)	
**Co-treatment**					
Low-flow oxygen, % (95% CI)	No	0.2 (0.0-0.9)	0.3 (0.1-0.7)	0.6 (0.3-1.0)	0.530
	Yes	Less than 3	1.5 (0.9-2.4)	1.3 (0.8-2.2)	
High-flow oxygen/NIV, % (95% CI)	No	0.8 (0.3-1.8)	1.4 (0.9-2.0)	1.2 (0.8-1.8)	0.567
	Yes	5.3 (2.9-9.0)	5.1 (3.9-6.5)	4.9 (3.8-6.3)	
Mechanical ventilation or EMCO, % (95% CI)	No	0.9 (0.3-2.0)	3.6 (2.8-4.5)	2.8 (2.1-3.6)	<0.001
	Yes	14.0 (9.9-19.0)	9.6 (7.9-11.4)	7.0 (5.6-8.6)	
Interleukin-6 inhibitor, % (95% CI)	No	Less than 3	Less than 3	1.0 (0.6-1.5)	0.962
	Yes	Less than 3	0.2 (0.0-0.6)	6.9 (5.6-8.5)	
Glucocorticoids, % (95% CI)	No	6.6 (4.9-8.8)	11.1 (9.7-12.6)	9.9 (8.6-11.3)	0.024

Characteristics	Remdesivir	First period*	Middle period*	Latest period*	P-value for interaction †
	Yes	34.2 (28.2-40.5)	39.5 (36.6-42.4)	45.2 (42.3-48.1)	
Antithrombotic medication, % (95% CI)	No	14.2 (11.6-17.2)	22.8 (20.9-24.8)	15.4 (13.9-17.1)	<0.001
	Yes	44.9 (38.5-51.3)	45.7 (42.7-48.6)	51.9 (49.0-54.7)	
Vasopressors, % (95% CI)	No	0.5 (0.1-1.3)	1.5 (1.0-2.2)	1.0 (0.6-1.6)	0.050
	Yes	2.1 (0.7-4.7)	1.0 (0.5-1.8)	1.3 (0.7-2.1)	
**Comorbidity‡**					
Ischemic heart disease, % (95% CI)	No	10.2 (8.0-12.8)	11.1 (9.7-12.7)	7.7 (6.6-9.0)	0.259
	Yes	7.0 (4.1-11.0)	10.8 (9.1-12.8)	8.5 (6.9-10.2)	
Kidney disease, % (95% CI)	No	6.3 (4.6-8.5)	8.9 (7.6-10.3)	5.3 (4.3-6.4)	0.784
	Yes	2.9 (1.2-5.8)	3.1 (2.2-4.3)	2.1 (1.4-3.1)	
Cancer, % (95% CI)	No	6.3 (4.6-8.5)	9.2 (8.0-10.7)	5.4 (4.5-6.5)	0.123
	Yes	6.2 (3.5-10.0)	10.8 (9.1-12.8)	8.7 (7.2-10.4)	
Immune deficiency, % (95% CI)	No	1.7 (0.9-3.0)	2.3 (1.7-3.1)	3.0 (2.3-3.9)	0.643
	Yes	Less than 3	1.8 (1.1-2.7)	1.7 (1.0-2.6)	
Hypertension, % (95% CI)	No	41.6 (37.7-45.5)	50.4 (48.1-52.7)	30.2 (28.1-32.3)	0.005
	Yes	51.0 (44.6-57.5)	53.6 (50.6-56.6)	41.0 (38.2-43.9)	
Diabetes, % (95% CI)	No	19.9 (16.9-23.2)	20.1 (18.3-22.0)	15.0 (13.4-16.6)	0.049
	Yes	24.3 (19.0-30.2)	27.9 (25.3-30.6)	25.7 (23.2-28.3)	
Chronic lung disease, % (95% CI)	No	21.9 (18.8-25.3)	22.7 (20.8-24.7)	19.1 (17.4-20.9)	0.250
	Yes	19.3 (14.6-24.9)	25.2 (22.7-27.9)	22.2 (19.8-24.6)	
Dementia, % (95% CI)	No	3.7 (2.4-5.5)	7.8 (6.6-9.2)	1.4 (0.9-2.0)	0.245
	Yes	2.9 (1.2-5.8)	6.4 (5.1-8.0)	1.9 (1.2-2.9)	
Heart failure, % (95% CI)	No	5.4 (3.8-7.4)	5.1 (4.2-6.2)	2.8 (2.1-3.6)	0.578
	Yes	4.5 (2.3-8.0)	5.3 (4.0-6.7)	3.4 (2.5-4.6)	
Atrial fibrillation, % (95% CI)	No	8.8 (6.7-11.3)	12.1 (10.7-13.7)	5.3 (4.4-6.4)	0.771
	Yes	7.4 (4.4-11.5)	12.2 (10.4-14.3)	4.9 (3.8-6.3)	
Stroke, % (95% CI)	No	3.1 (1.9-4.7)	7.2 (6.1-8.5)	3.0 (2.3-3.9)	0.248
	Yes	3.3 (1.4-6.4)	5.9 (4.6-7.4)	3.7 (2.7-4.9)	
Chronic liver disease, % (95% CI)	No	1.1 (0.4-2.2)	1.8 (1.2-2.5)	1.5 (1.0-2.1)	0.433
	Yes	2.1 (0.7-4.7)	1.5 (0.9-2.4)	1.3 (0.7-2.1)	

Notes:
*The columns "First period", "Middle period", and "Latest period" are presented as "mean (95% CI)" unless otherwise stated.
†The p-value for the interaction is based on a logistic regression model with remdesivir treatment as the outcome and the interaction between the variable and the time period.
‡From 2 years before hospitalization until and including the day of hospitalization.
CI – Confidence interval

Checklist S1. STROBE Statement—Checklist of items that should be included in reports of ***cohort studies***

	Item No	Recommendation	Page No
**Title and abstract**	1	(*a*) Indicate the study's design with a commonly used term in the title or the abstract	1
		(*b*) Provide in the abstract an informative and balanced summary of what was done and what was found	2
**Introduction**			
Background/rationale	2	Explain the scientific background and rationale for the investigation being reported	3
Objectives	3	State specific objectives, including any prespecified hypotheses	4
**Methods**			
Study design	4	Present key elements of study design early in the paper	4
Setting	5	Describe the setting, locations, and relevant dates, including periods of recruitment, exposure, follow-up, and data collection	4
Participants	6	(*a*) Give the eligibility criteria, and the sources and methods of selection of participants. Describe methods of follow-up	4/5
		(*b*) For matched studies, give matching criteria and number of exposed and unexposed	
Variables	7	Clearly define all outcomes, exposures, predictors, potential confounders, and effect modifiers. Give diagnostic criteria, if applicable	5/6
Data sources/ measurement	8*	For each variable of interest, give sources of data and details of methods of assessment (measurement). Describe comparability of assessment methods if there is more than one group	4/5/6
Bias	9	Describe any efforts to address potential sources of bias	6
Study size	10	Explain how the study size was arrived at	4
Quantitative variables	11	Explain how quantitative variables were handled in the analyses. If applicable, describe which groupings were chosen and why	6/7
Statistical methods	12	(*a*) Describe all statistical methods, including those used to control for confounding	6/7
		(*b*) Describe any methods used to examine subgroups and interactions	6/7
		(*c*) Explain how missing data were addressed	6/7
		(*d*) If applicable, explain how loss to follow-up was addressed	NA
		(*e*) Describe any sensitivity analyses	NA
**Results**			
Participants	13*	(a) Report numbers of individuals at each stage of study—eg numbers potentially eligible, examined for eligibility, confirmed eligible, included in the study, completing follow-up, and analysed	7
		(b) Give reasons for non-participation at each stage	7
		(c) Consider use of a flow diagram	7

Descriptive data	14*	(a) Give characteristics of study participants (eg demographic, clinical, social) and information on exposures and potential confounders<br>(b) Indicate number of participants with missing data for each variable of interest<br>(c) Summarise follow-up time (eg, average and total amount)	8<br>Supplementary<br>5/6/7
Outcome data	15*	Report numbers of outcome events or summary measures over time	8/9

Main results	16	(*a*) Give unadjusted estimates and, if applicable, confounder-adjusted estimates and their precision (eg, 95% confidence interval). Make clear which confounders were adjusted for and why they were included<br>(*b*) Report category boundaries when continuous variables were categorized<br>(*c*) If relevant, consider translating estimates of relative risk into absolute risk for a meaningful time period	7-9<br>6/7<br>NA
Other analyses	17	Report other analyses done—eg analyses of subgroups and interactions, and sensitivity analyses	6/7/supplementary
**Discussion**			
Key results	18	Summarise key results with reference to study objectives	10
Limitations	19	Discuss limitations of the study, taking into account sources of potential bias or imprecision. Discuss both direction and magnitude of any potential bias	14
Interpretation	20	Give a cautious overall interpretation of results considering objectives, limitations, multiplicity of analyses, results from similar studies, and other relevant evidence	11/12/13
Generalisability	21	Discuss the generalisability (external validity) of the study results	12/13/14
**Other information**			
Funding	22	Give the source of funding and the role of the funders for the present study and, if applicable, for the original study on which the present article is based	16
